# Physiology and Pharmacology of DPP-4 in Glucose Homeostasis and the Treatment of Type 2 Diabetes

**DOI:** 10.3389/fendo.2019.00080

**Published:** 2019-02-15

**Authors:** Carolyn F. Deacon

**Affiliations:** Department of Biomedical Sciences, University of Copenhagen, Copenhagen, Denmark

**Keywords:** dipeptidyl peptidase-4, glucagon-like peptide-1, incretin, peptide degradation, therapy, type 2 diabetes

## Abstract

Dipeptidyl peptidase-4 (DPP-4), also known as the T-cell antigen CD26, is a multi-functional protein which, besides its catalytic activity, also functions as a binding protein and a ligand for a variety of extracellular molecules. It is an integral membrane protein expressed on cells throughout the body, but is also shed from the membrane and circulates as a soluble protein in the plasma. A large number of bioactive molecules can be cleaved by DPP-4 *in vitro*, but only a few of these have been demonstrated to be physiological substrates. One of these is the incretin hormone, glucagon-like peptide-1 (GLP-1), which plays an important role in the maintenance of normal glucose homeostasis, and DPP-4 has been shown to be the key enzyme regulating its biological activity. This pathway has been targeted pharmacologically through the development of DPP-4 inhibitors, and these are now a successful class of anti-hyperglycaemic agents used to treat type 2 diabetes (T2DM). DPP-4 may additionally influence metabolic control via its proteolytic effect on other regulatory peptides, but it has also been reported to affect insulin sensitivity, potentially mediated through its non-enzymatic interactions with other membrane proteins. Given that altered expression and activity of DPP-4 are associated with increasing body mass index and hyperglycaemia, DPP-4 has been proposed to play a role in linking obesity and the pathogenesis of T2DM by functioning as a local mediator of inflammation and insulin resistance in adipose and hepatic tissue. As well as these broader systemic effects, it has also been suggested that DPP-4 may be able to modulate β-cell function as part of a paracrine system involving GLP-1 produced locally within the pancreatic islets. However, while it is evident that DPP-4 has the potential to influence glycaemic control, its overall significance for the normal physiological regulation of glucose homeostasis in humans and its role in the pathogenesis of metabolic disease remain to be established.

## Introduction

**Figure 1:**
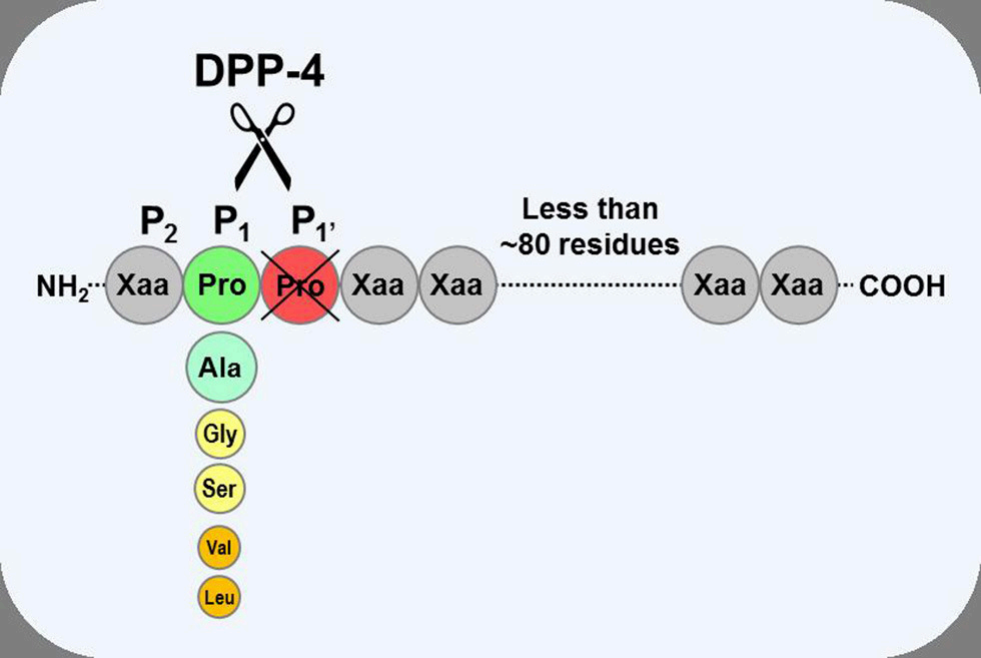
Substrate specificity of DPP-4. DPP-4 is an amino peptidase which liberates a dipeptide from its substrates. It prefers peptides or small proteins (below 80–100 residues) with proline or alanine as the penultimate N-terminal residue, although some substrates with glycine, serine, valine, or leucine can be cleaved at a slower rate. The enzyme is unable to cleave substrates with proline in position three.

**Figure 2:**
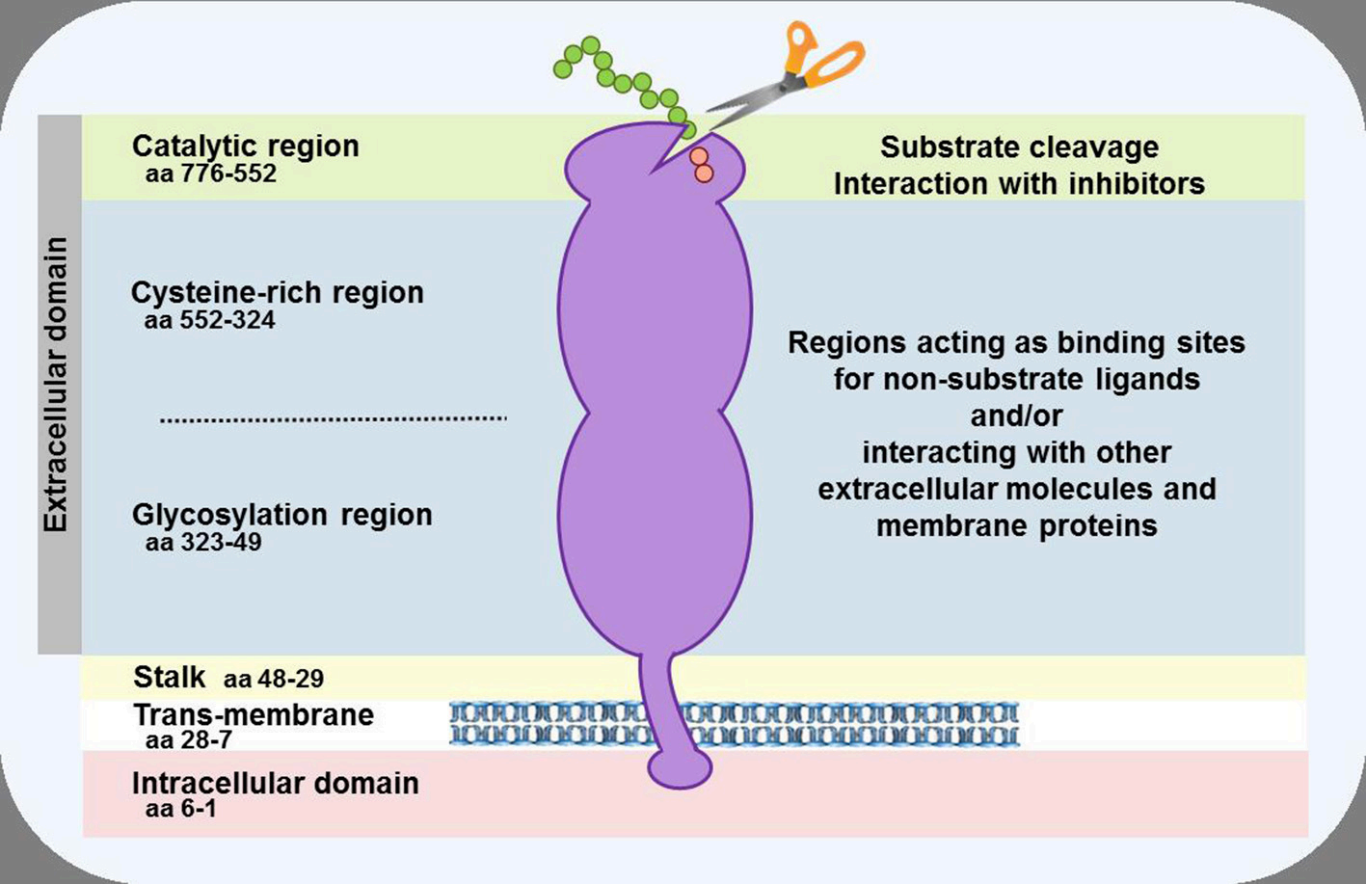
Schematic representation of the DPP-4 protein. In the cell membrane, two DPP-4 monomers dimerise to form a homodimer. The monomers can be cleaved at the stalk to release the soluble form of DPP-4, which circulates in the plasma. The enzymatic activity resides in the catalytic pocket, formed by residues (including serine at position 630) located in the C-terminal portion of the protein. Sites within the cysteine-rich and glycosylation regions serve as a receptor or ligand for different molecules, including adenosine deaminase, caveolin-1, collagen, fibronectin, chemokine CXCR4 receptor, CD45, and the sodium-hydrogen exchanger-3, to mediate the non-enzymatic functions of the protein.

Dipeptidyl peptidase-4 (DPP-4; EC 3.4.14.5) is a member of the prolyl oligopeptidase family of related proteins, which, besides prolyl oligopeptidase (a.k.a. prolyl endopeptidase), contains a number of exopeptidases, including quiescent cell proline dipeptidase (QPP, a.k.a. DPP-II or DPP-7), and attractin (a.k.a. mahogany peptide). Within this family, the DPP-4 gene family belongs to the subclan S9B, and comprises six proteins, of which four [fibroblast activation protein (FAPα, a.k.a. seprase), DPP-4, -8, and -9] are enzymatically active, but also exert effects via protein-protein interactions, and two [DPP-6, known also as DPP4-like protein (DPL)-1] and DPP-10 (a.k.a. DPL-2) are catalytically inactive, exerting their effects by modulating voltage-gated potassium channels (Table 1) (1–3). DPP-4 is an amino-peptidase, which liberates a dipeptide from the N-terminal of its substrates. Like other members of the family, it prefers proline or alanine in the penultimate position, although peptides with other residues (glycine, serine, valine) in this position can be cleaved, albeit more slowly (Figure 1). DPP-4 is an integral membrane protein which has a widespread distribution, being expressed in numerous tissues including intestinal and renal brush border membranes, vascular endothelium, the liver and pancreas, glandular epithelial cells, and by cells of the immune system (where it is also known as the T-cell differentiation antigen, CD26). The DPP-4 protein consists of a large extracellular domain, anchored in the cell membrane by a flexible segment coupled to a trans-membrane sequence, with a short intracellular tail at the N-terminus (Figure 2). It can, however, be cleaved at the stalk to release the extracellular domain, which circulates in the plasma as “soluble” DPP-4 (aa 49-766), but is also present in other bodily fluids such as seminal fluid and cerebrospinal fluid. The catalytic site resides in the C-terminal region of the extracellular part of the protein, but the extracellular domain also contains a cysteine-rich region and a region rich in glycosylation sites. These regions are believed to be involved in many of the non-enzymatic functions of DPP-4, interacting with other proteins, such as adenosine deaminase, caveolin-1, streptokinase and plasminogen, and with components of the extracellular matrix (collagen, fibronectin), as well as functioning as binding sites for the chemokine CXCR4 receptor, the T-cell differentiation antigen, CD45, and the sodium-hydrogen exchanger-3, among others (1, 4).

**Table 1:** The proline-specific peptidases.

	**Catalytic**	**Location**	**Distribution**	
QPP	✓	Cytoplasmic	Quiescent T-cells, placenta	
DPP-4	✓	Transmembrane soluble	Ubiquitous (epithelial, endothelial, immune cells)	
DPP-6	✗	Transmembrane	Brain (prostate, kidney, testis)	DPP-4 gene family
DPP-8	✓	Cytoplasmic	Ubiquitous	
DPP-9	✓	Cytoplasmic	Ubiquitous	
FAPα	✓	Transmembrane (soluble)	Activated fibroblasts and hepatic stellate cells	
DPP-10	✗	Transmembrane	Brain, pancreas	
PEP	✓	Cytoplasmic soluble	Ubiquitous	
Attractin	✓	Transmembrane soluble	Brain, T-cells, placenta, liver, kidney, plasma	

*QPP, quiescent cell proline dipeptidase; DPP, dipeptidyl peptidase; FAP, fibroblast activation protein; PEP, prolyl endopeptidase*.

Accordingly, DPP-4 can be viewed as a multi-functional protein with a spectrum of actions which go beyond its role as a proteolytic enzyme. There is a wealth of data identifying DPP-4 as a receptor or ligand for a variety of different molecules which, either alone or in combination with its enzymatic activity, allow it affect physiological processes such as the interaction between cells and the extracellular matrix, cell migration, and proliferation. Within the immune system, DPP-4/CD26 is one of several proteins which can act as co-stimulatory molecules. These molecules cannot directly stimulate T-cells, but they are involved in amplifying the signal derived from the interaction with an antigen via their interaction with other cell surface molecules, thereby leading to T-cell activation (5). For DPP-4, this immune function appears to be independent of its catalytic activity, since the co-stimulatory activity of the molecule is retained by mutant proteins lacking the catalytic region (6) and is unaffected by inhibitors of the enzymatic activity of DPP-4 (7). Consequently, given its various roles, altered expression, and/or activity of DPP-4 have been implicated in several pathological processes, including inflammation, viral entry, immune-mediated diseases, and tumor biology (1, 3, 4). More recently, DPP-4 has been found to have effects on metabolic control, raising the possibility that it may play a role in metabolic diseases such as diabetes and obesity. However, the relative importance of the soluble vs. the membrane-bound form of the molecule for the various functions of DPP-4 has not been clarified, nor is it clear whether (or to what extent) changes in circulating levels of soluble DPP-4 might be regulated (e.g., whether they simply reflect alterations in non-specific cleavage or shedding of the membrane-bound form or whether this is a process which might be modulated in response to pathological processes or changes in metabolic status).

## DPP-4 and Glucose Homeostasis

The link between DPP-4 and glucose homeostasis was only identified after the intestinal hormone, glucagon-like peptide-1 (GLP-1) was found to be a DPP-4 substrate. A role for GLP-1 in regulating glycaemia had been noted in 1986, when this, then newly described (8, 9), peptide was found to have profound effects upon the endocrine pancreas. In studies using perfused pig and rat pancreata, researchers in Denmark and the US described potent insulinotropic (10, 11), and glucagonostatic (12) effects *in vitro*, while studies in healthy humans revealed that circulating levels of GLP-1 increased after nutrient intake and confirmed GLP-1 to be an incretin hormone *in vivo* (13). However, it was when the insulinotropic and glucose-lowering effects were shown to be preserved in patients with type 2 diabetes (T2DM) (14, 15) that real interest in GLP-1 took off. In a now landmark study, Michael Nauck demonstrated that hyperglycaemic fasting glucose levels could be brought into the normal range by intravenous GLP-1 infusion in individuals with T2DM (16). Moreover, this study also nicely illustrated the glucose dependency of GLP-1's anti-diabetic actions—insulin secretion was stimulated, and glucagon secretion suppressed, only at elevated glucose levels, with the effects of GLP-1 becoming progressively less as euglycaemia was approached. However, whilst intravenous infusion of GLP-1 was effective (16), the insulinotropic effect of a single subcutaneous injection was surprisingly short-lived, with insulin levels peaking at 30 min before returning toward baseline within 90 min, even though glucose levels were still well within the hyperglyacemic range and circulating immunoreactive GLP-1 levels were significantly elevated for several hours (17). The reason for this paradox was initially unclear. However, when it was reported that GLP-1 was a substrate for DPP-4 in pharmacological *in vitro* kinetic studies (18), this was quickly followed by the demonstration that the metabolite generated by DPP-4 cleavage was the major circulating component of GLP-1-like immunoreactivity in healthy individuals (19) and that the same metabolite formed rapidly following exogenous administration of GLP-1 in both healthy subjects and those with T2DM (20). In line with these results, similar findings were reported after exogenous GLP-1 administration in rats (21). These studies, therefore, indicated that GLP-1 was a true physiological substrate of DPP-4, and led to the suggestion that blocking this route of degradation may be a way to increase endogenous intact (active) GLP-1 concentrations and enhance its anti-hyperglycaemic actions (20). Accordingly, and using the analogy of angiotensin-converting enzyme inhibitors for treating hypertension, DPP-4 inhibition was proposed as a novel approach to the treatment of T2DM (20, 22).

## Inhibiting DPP-4 as a Therapy for T2DM

In order for this approach to be viable, DPP-4 cleavage would need to be the initial and primary route of metabolism of GLP-1. If this was not the case, other clearance pathways would simply take over once the DPP-4 pathway had been blocked, and levels of the intact peptide would not be increased. In those early days, several DPP-4 inhibitors had been described (23), but none were suitable for human use, so proof-of-hypothesis came from preclinical studies. In anesthetized pigs, a prototype DPP-4 inhibitor, valine pyrrolidide, was demonstrated to reduce plasma DPP-4 activity sufficiently to fully protect intravenously infused GLP-1 from degradation. Moreover, this was associated with an enhanced insulin response, confirming that DPP-4-mediated degradation played a significant role in limiting the insulinotropic effect of GLP-1 (24). The pivotal role of DPP-4 in endogenous GLP-1 metabolism was highlighted using the isolated perfused porcine small intestine, which revealed that over half of newly released GLP-1 was degraded even before it left the splanchnic bed. Again, this could be completely prevented by DPP-4 inhibition with valine pyrrolidide, confirming that cleavage by DPP-4 was the key initial step in GLP-1 degradation, and that other enzymatic pathways played a limited role (25). This study also investigated the expression of DPP-4 and found it to be present on the vascular endothelium, including the local capillaries in the lamina propria adjacent to the GLP-1 producing L-cells, thus providing an explanation for the rapid degradation of the peptide once it had been released. An acute glucose-lowering effect of DPP-4 inhibition in a rodent model of T2DM (the obese Zucker rat) was demonstrated when it was shown that a different inhibitor (isoleucine thiazolidide) reduced plasma DPP-4 activity and was associated with a larger insulin response and improved glucose tolerance after an oral glucose load (when endogenous GLP-1 secretion would be stimulated) (26). No effects were seen when glucose was not administered, compatible with the idea that DPP-4 inhibition exerts an anti-hyperglycaemic effect by preventing degradation of endogenously released GLP-1 (20). Although GLP-1 levels were not actually measured in that study (26), subsequent studies in glucose-intolerant rodent models did show that the improved glycaemic control following *in vivo* DPP-4 inhibition was associated with enhanced intact GLP-1 responses to glucose challenges (27–29). The final step in establishing preclinical proof-of-hypothesis was made when the results of these acute studies were recapitulated with chronic dosing, showing that the beneficial pancreatic islet and glucose-lowering effects of DPP-4 inhibition persisted over several months of treatment (30, 31). Collectively, these studies paved the way for clinical investigation into the feasibility of using pharmacological inhibition of DPP-4 to improve glycaemic control in patients with T2DM. The first two reports, both 4 week studies in drug naïve patients with relatively mild T2DM, showed that DPP-4 inhibition was well-tolerated in humans and was associated with significantly lower fasting and meal-related glucose concentrations (32, 33). Absolute insulin responses were not augmented, but they were sustained in face of the improved glycaemia which, together with the lower glucagon levels could be suggestive of an improved islet function related to the observed increase in intact GLP-1 levels (33). The longer-term efficacy of DPP-4 inhibition was examined in a third study published later in the same year, this time undertaken in patients with T2DM on metformin monotherapy. This 12 week study with a 40 week extension period revealed that the addition of vildagliptin significantly lowered HbA1c levels by week 12, with the improvement being sustained to the end of the study period, whereas glycaemic control deteriorated in the placebo recipients (34). As in the shorter-term studies, DPP-4 inhibition was well-tolerated with no increased risk of hypoglycaemia, despite the improvement in glycaemic control, and the overall adverse event profile was similar in both arms of the study. These key studies clearly provided clinical proof-of-concept that blocking the catalytic activity of DPP-4 could be a viable approach to treat T2DM, whilst at the same time helped to allay concern over whether the metabolism of any other potential DPP-4 substrates might be affected to cause unwanted off-target effects. These studies were soon followed by a number of phase 2 and phase 3 clinical trials with several different DPP-4 inhibitors, all essentially showing that DPP-4 inhibition was a safe and well-tolerated means of improving glycaemic control in patients with T2DM. The first DPP-4 inhibitor received marketing authorization in 2006, and now, world-wide, there are currently at least 11 different inhibitors approved for therapy of T2DM (35).

## Metabolic Effects of DPP-4 Related to Its Enzymatic Activity

Despite DPP-4 having a relatively restricted substrate preference (Figure 1), a large number of peptides, proteins, and chemokines still fulfill the criteria and could be predicted to be suitable substrates, and indeed, many have been shown to be cleaved by DPP-4 in *pharmacological* kinetic, *in vitro* or animal studies, often using high substrate concentrations (1, 4, 36–38). However, simply showing that the enzyme *can* cleave a substrate *in vitro* does not necessarily mean that DPP-4 plays an important physiological role in regulating concentrations and/or modulating biological activity of the *endogenous* molecules, particularly in humans and, for many of these putative substrates, there is little, if any experimental evidence to show that DPP-4 does actually cleave them *in vivo* and/or whether this has any physiological consequence. Thus, for a number of them, even if they are good substrates *in vitro*, other clearance pathways may be more important *in vivo* or, if they are cleaved by DPP-4, removal of the N-terminal dipeptide may have no effect upon their biological activity. However, it should also be recognized that it may be difficult to definitively demonstrate whether DPP-4 does play a physiologically relevant role in cleaving some endogenous substrates *in vivo* because it can be difficult to distinguish between the intact substrate and the truncated metabolite (because assays are not specific or sensitive enough) and/or because it may be difficult to obtain appropriate samples (e.g., from synapses [neurotransmitters] or interstitial fluid [paracrine signallers]). Accordingly, the list of molecules which have been shown unequivocally to be physiological substrates of DPP-4 is short (37, 38). Moreover, given that there are now millions of patient-years of clinical experience with DPP-4 inhibitors since they were given regulatory approval in 2006, their benign, placebo-like side effect profile (39) also suggests that the number of physiologically relevant substrates is likely to be limited.

### GLP-1 and GIP

With regard to metabolic effects, the best characterized DPP-4 substrates are the two incretin hormones, GLP-1, and glucose-dependent insulinotropic polypeptide (GIP). As discussed above, endogenous GLP-1 is rapidly degraded by DPP-4, whereby it loses its insulinotropic activity, and preventing this degradation results in increased intact GLP-1 levels, improved pancreatic islet responses (enhanced insulin and suppressed glucagon levels) and beneficial effects on glucose homeostasis. Similarly, the other incretin hormone, GIP is efficiently cleaved by DPP-4 *in vivo* (40), and DPP-4 inhibition increases intact GIP levels and enhances its effects (41). Accordingly, it is well-established that DPP-4 does play a pivotal role in the initial inactivation of both endogenous incretin hormones. It is, however, less clear whether this is a process which can be regulated, or whether it is simply a consequence of DPP-4's ubiquitous distribution. Nevertheless, the fact that DPP-4 is a key player in the clearance of both endogenous peptides has been exploited pharmacologically and, as mentioned above, DPP-4 inhibitors are now an established therapy of T2DM.

It was initially thought that the anti-diabetic effects of DPP-4 inhibition were predominantly due to the enhanced levels of intact GLP-1 [because the insulinotropic effect of GIP is substantially impaired in subjects with T2DM (42)], but this now seems not to be the case. Thus, in mice, significant antihyperglycaemic effects of DPP-4 inhibition are still apparent in animals lacking the GLP-1 receptor (43), while in clinical studies, GLP-1 receptor antagonism (using exendin 9-39) eliminates only approximately half of the glucose-lowering effect in patients with T2DM treated with DPP-4 inhibitors (44, 45). It is possible that GIP may still be involved in spite of the observations that its actions are impaired in patients with T2DM (42), because it appears that these can be at least partially restored. Thus, treatment to improve glycaemic control, using insulin (46) or a DPP-4 inhibitor (47) led to improved insulin responses to exogenous GIP. This would be in line with preclinical observations that incretin receptor expression is down-regulated by exposure to high glucose, and improves once normoglycaemia is restored (48), and that dys-regulation of glycaemic control in otherwise healthy humans leads to impaired incretin actions (49). Thus, GIP is a likely candidate for mediating some of the therapeutic actions of DPP-4 inhibition, and the recent development of a GIP receptor antagonist suitable for use in clinical studies (50) should allow this hypothesis to be tested.

### Peptide Tyrosine Tyrosine

Peptide tyrosine tyrosine (PYY) is another gastrointestinal peptide which is released after food ingestion and which also fulfills the criteria to be classified as a physiological DPP-4 substrate. Both exogenous and endogenous PYY have been shown to be degraded *in vivo* in animal and clinical studies. Moreover, the truncated peptide, PYY 3-36, is the major circulating endogenous form in humans (51), and its formation is reduced by DPP-4 inhibition (52). In contrast to the incretin hormones, where cleavage by DPP-4 results in a loss of their insulinotropic activity, cleavage of PYY results in altered receptor selectivity. Thus, intact PYY 1-36 binds with equal affinity to the four Y receptor subtypes, while PYY 3-36 is a selective high affinity agonist for the Y2 receptor. Although there is no evidence that PYY can directly influence insulin secretion in humans (53), activation of Y1 receptors is associated with increased intestinal motility and orexigenic effects, while conversely, Y2 receptor activation mediates the enterogastrone and anorectic effects of PYY (54). Accordingly, DPP-4 may influence metabolic control indirectly by modulating PYY effects on food intake and body weight. Again, whether this is a process which is regulated is unknown, but it may help to explain why pharmacological use of DPP-4 inhibitors is generally not associated with weight loss (the appetite suppressive effects of increased intact GLP-1 levels being counter-balanced by the loss of the anorectic effects of PYY 3-36).

### Oxyntomodulin

Surprisingly, given that glucagon is not a physiological DPP-4 substrate (37, 38), there is some evidence that the C-terminally extended peptide, oxyntomodulin might be. Oxyntomodulin is generated by post-translational processing of the glucagon precursor, proglucagon, in the intestine. A specific oxyntomodulin receptor has not been identified, and oxyntomodulin is believed to exert its effects by behaving as a weak agonist at both the GLP-1 and glucagon receptors (55). Accordingly, it has several metabolic effects consistent with the actions of GLP-1 and glucagon (stimulation of insulin secretion, reduced appetite, increased energy expenditure), although whether these are important physiologically is uncertain (55). Nevertheless, oxyntomodulin can be cleaved by DPP-4 *in vitro* (36, 56), and in animal studies, the anorectic effects of exogenous oxyntomodulin are increased after DPP-4 inhibition (56), while DPP-4-resistant oxyntomodulin analogs have been associated with improved glucose tolerance, reduced food intake, greater energy expenditure, and weight loss (57, 58). Nevertheless, because endogenous oxyntomodulin is currently not believed to play a major role in regulating metabolic processes (55), it seems unlikely that cleavage of oxyntomodulin by DPP-4 would be associated with significant effects under normal physiological circumstances. However, whether pharmacological inhibition of the enzyme could elevate oxyntomodulin levels sufficiently to contribute to the therapeutic effect of DPP-4 inhibitor therapy is not known.

### Stromal Cell-Derived Factor-1α

There is fairly strong evidence that stromal cell-derived factor-1 (SDF-1α) also qualifies as a physiological DPP-4 substrate (37, 38). SDF-1α, also referred to as CXCL12, functions as a chemotactic cytokine and proangiogenic chemokine, which enhances hematopoietic and endothelial progenitor cell recruitment to sites of cell injury. However, it is also expressed in the pancreas, where *in vitro* studies have indicated that it can improve β-cell survival (59, 60) and enhance α-cell GLP-1 biosynthesis (60). Moreover, transgenic mice over-expressing SDF-1α become resistant to streptozotocin-induced diabetes (61), further suggesting that SDF-1α may have some metabolic effects. The involvement of DPP-4 in metabolism of endogenous SDF-1α is compatible with the finding that the truncated metabolite, SDF-1α 3-67, was present in wild-type mice, but not in animals lacking DPP-4 (62), with this observation being extended in subsequent studies in mice and Rhesus monkeys (63) and in patients with T2DM (64) showing that levels of SDF-1α 3-67 were reduced while those of the intact 1-67 form increased concomitantly when DPP-4 activity was inhibited. Cleavage of SDF-1α by DPP-4 results in loss of activity (65), and consistent with this, DPP-4 inhibition in humans has been associated with enhancement of some effects which are associated with SDF-1α [increased levels of circulating endothelial progenitor cells (66) and increased distal tube sodium excretion (64)]. However, although the preclinical studies cited above point toward potential metabolic effects of SDF-1α, it is unknown whether there are any physiological or pharmacological consequences of DPP-4-mediated cleavage of SDF-1α for metabolic control in humans.

## A Potential Role for DPP-4 in the Pathogenesis of T2DM?

Given that DPP-4 clearly plays a role in the clearance of a number of hormones involved in the regulation of glucose homeostasis, the question arises of whether changes in DPP-4 activity might play a role in the pathogenesis of T2DM. Indeed, a number of reports have included assessment of DPP-4 activity, albeit with conflicting results. While some studies found no difference between individuals with T2DM and matched non-diabetic controls (67–69), others found plasma DPP-4 activity to be reduced in the patients, and suggested this might reflect a compensatory response to the reduced insulin sensitivity and hyperglycaemia, in an attempt to increase intact incretin hormone concentrations and improve insulin secretion (70, 71). However, increased levels have also been reported (68, 72, 73), raising speculation that elevated plasma DPP-4 activity might constitute a risk factor associated with the deterioration of glycaemic control. In this scenario, greater degradation of GLP-1 (and the other incretin, GIP), with a consequent impairment of the incretin effect would contribute to worsening of glycaemic control. This might arise early in the pathogenesis of T2DM, as one of many risk factors contributing to the initial onset of hyperglycaemia in susceptible individuals, or later, as a consequence of already existing metabolic changes causing increased DPP-4 activity and further exacerbating the deterioration of glycaemic control. Accordingly, several studies have reported a positive correlation between plasma DPP-4 activity and fasting plasma glucose or HbA1c (68, 72), although it should be borne in mind that an association does not necessarily reflect a causative relationship. Moreover, it appears that it is the membrane-bound form of the enzyme, on sites such as the luminal surface of capillary endothelial cells, which is more important for GLP-1 degradation than the circulating (soluble) enzyme (74, 75), questioning the relevance of any association between the plasma activity *per se* and glycaemic control, since it is unclear whether changes in soluble DPP-4 activity in the circulation accurately reflect any changes in total DPP-4 activity. Glucose itself does not seem to directly modulate the catalytic activity of DPP-4, as shown *in vitro*, by incubating the enzyme with high concentrations of glucose (76). Moreover, plasma DPP-4 activity is not lowered when glycaemic control is improved in patients with T2DM, thereby also suggesting that hyperglycaemia is not a direct determinant of levels of DPP-4 activity (73). However, exposure to high glucose conditions did increase mRNA expression and membrane-bound DPP-4 activity in human glomerular (76) and dermal (77) microvascular endothelial cells *in vitro*, although, interestingly, not in macrovascular (human aortic) endothelial cells (77). Changes in levels of plasma DPP-4 activity could, therefore, be related to overall changes in the total expression of DPP-4 and/or simply altered shedding from cell membranes. Nonetheless, the underlying reasons for the inconsistencies in the reported associations between T2DM and levels of plasma DPP-4 activity (lower, unaltered, higher) have not been adequately explained, although one study did suggest that it might be related to the populations studied, with changes only becoming evident once moderate or severe hyperglycaemia becomes established (68).

On the other hand, T2DM is often associated with increased body mass index (BMI), and other studies have suggested that levels of plasma DPP-4 activity actually correlate better with clinical parameters of obesity rather than of T2DM and glucose *per se*. Thus, a number of studies have shown a positive association between plasma DPP-4 activity and BMI in non-diabetic individuals (78–80), and it has been proposed that the release of DPP-4 from adipose tissue may provide one explanation for the increased plasma DPP-4 activity measured in obese subjects. Accordingly, the DPP-4 protein has been identified in both subcutaneous and visceral adipose tissue, with its expression, particularly in visceral adipocytes, being more pronounced in obese subjects (81). In another study, both circulating DPP-4 levels and the amount released from adipocyte explants *in vitro* were found to be positively correlated with adipocyte size as well as with anthropometric parameters such as BMI, waist circumference and percent body fat (79). Moreover, following surgically-induced weight loss in morbid obesity, the elevated adipocyte DPP-4 release, and circulating DPP-4 levels were both substantially reduced, further suggesting that the adipose tissue is likely to be a major source of plasma DPP-4 (79). On the other hand, *Dpp4* expression is reported to be relatively low in mouse adipocytes and not to be markedly increased after high fat feeding (82), with expression within adipose tissue being predominantly associated with cells of the stromal vascular fraction (82, 83).

However, there is also evidence pointing toward a hepatic origin of circulating DPP-4, and it has been suggested that the inclusion of some patients with unidentified liver disease may have contributed to the conflicting data regarding the association of plasma DPP-4 activity and T2DM. In mice, hepatocyte *Dpp4* expression increases with obesity (82), and selective elimination of *Dpp4* in the liver is associated with reduced levels of soluble DPP-4 in the plasma (82, 83). In humans, plasma DPP-4 activity is higher in patients with non-alcoholic fatty liver disease irrespective of their glucose tolerance, whereas it was not elevated in patients with T2DM in whom liver disease had been excluded (84). This association between liver disease and DPP-4 has been confirmed in other studies, showing higher plasma levels (85, 86) as well as higher hepatic *Dpp4* expression (87) in the patients compared to healthy individuals.

## Protease-Independent Metabolic Effects of DPP-4

**Figure 3:**
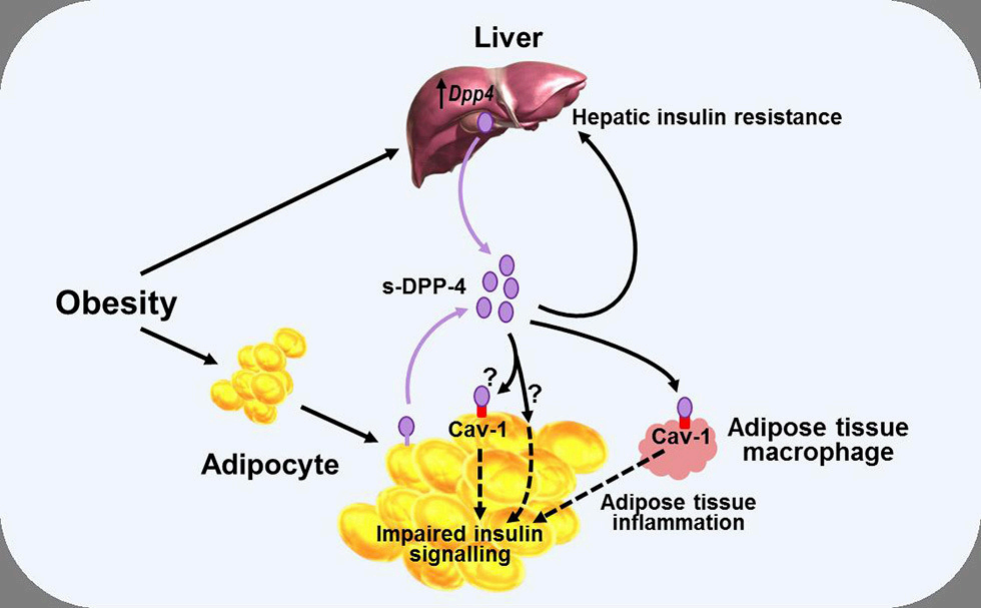
Diagram illustrating a potential role of DPP-4 as a mechanism linking obesity with inflammation and insulin resistance. Obesity is associated with increased levels of soluble DPP-4 (s-DPP-4), derived from up-regulated hepatocyte *Dpp4* expression and larger adipocytes shedding more DPP-4 from the cell membrane. Increased levels of soluble DPP-4 result in adipocyte insulin resistance, possibly mediated via interactions with caveolin-1 (Cav-1) expressed on the cell surface of adipocytes themselves and on adipose tissue macrophages. Increased soluble DPP-4 levels are also associated with increased hepatic insulin resistance. Reduced insulin sensitivity in adipose and hepatic tissue may lead to hyperglycaemia, further exacerbating insulin resistance. See text for further details.

While some of the effects of DPP-4—and, arguably, most, if not all of the pharmacological effects of DPP-4 inhibitors (38)—on glucose homeostasis are mediated primarily by modulating the activity of the incretin hormones, it has also been proposed that DPP-4 may influence metabolic control via mechanisms which do not necessarily involve its catalytic activity, with several studies noting a positive correlation between DPP-4 and insulin resistance (79, 81, 88). Accordingly, DPP-4 has been proposed to function as a locally acting adipokine with influence on insulin sensitivity. *In vitro* studies have shown that addition of DPP-4 is associated with impaired insulin signaling in primary human adipocytes, skeletal muscle, and smooth muscle cells (79), while conversely, silencing *Dpp4* (using short interfering RNA) resulted in improved adipocyte insulin responsiveness (89). In both cases, the mechanism was suggested to involve the insulin receptor and downstream signaling pathways including protein kinase B (Akt), although how DPP-4 might influence this process was not clarified. The catalytic activity of the molecule was suggested to be, at least partially, involved (since insulin signaling was improved following inclusion of a DPP-4 inhibitor), but the DPP-4 substrate(s) mediating the effect were not identified. DPP-4 can also have signaling functions via interactions with the extracellular matrix (90), and it was therefore speculated that the DPP-4 inhibitor might have improved insulin sensitivity by interfering with this process in some way rather than by blocking the enzymatic activity *per se* (79, 89). However, DPP-4 can interact with the integral membrane protein, caveolin-1 (91), which is expressed in various cell types, including adipocytes (92), and may be upregulated in obesity (93). In adipocytes, caveolin-1 plays a role in the insulin transduction pathway by modulating the activity of down-stream signaling proteins including Akt (94), raising the possibility that DPP-4 may be able to influence adipocyte insulin sensitivity by modulating the activity of caveolin-1 (Figure 3).

In an analogous manner, DPP-4 has been linked with hepatic insulin sensitivity in several studies. Thus, in mice, hepatocyte-specific overexpression of *Dpp4* is associated with hepatic insulin resistance and liver steatosis (86), whereas knockdown of *Dpp4* results in improved insulin sensitivity and reduced lipid accumulation in cultured hepatocytes (95). Yet other studies have pointed toward DPP-4 acting as a hepatokine, linking the liver and adipose tissue with the development of insulin resistance, and glucose intolerance. In mice, obesity and the associated visceral adipose tissue inflammation result in insulin resistance, a process which appears to be mediated via increased synthesis and release of hepatic DPP-4, since eliminating hepatocyte *Dpp4* expression suppresses inflammation and improves insulin sensitivity (82, 83). The mechanism seems to be independent of the catalytic activity, since these effects were not mimicked by systemic DPP-4 inhibition (73, 82), and it was suggested that soluble DPP-4 interacted with caveolin-1 on adipose tissue macrophages, the down-stream effects of which combined with actions of other pro-inflammatory molecules such as factor Xa, to lead to activation of the inflammatory signaling pathway (83) (Figure 3). However, it has also been reported that although deletion of hepatocyte *Dpp4* expression abolished the obesity-induced rise in circulating DPP-4 and was associated with reduced levels of proinflammatory cytokines in hepatocytes and adipose tissue, levels of circulating soluble DPP-4 *per se* did not correlate with tissue or systemic inflammation (82). Accordingly, the role of hepatocyte DPP-4 and the precise relationship between soluble DPP-4 and inflammation is complex, and the mechanisms linking hepatocyte *Dpp4* expression with inflammation remain to be clarified.

Nevertheless, taken together, these studies indicate that DPP-4 may be able to influence fat and liver biology via both direct and indirect pathways, and suggest, therefore, that metabolic control could be perturbed if DPP-4 levels change. As discussed above, DPP-4 levels are positively correlated with BMI and insulin resistance and, in some studies, also to glycaemic control, but the mechanisms regulating synthesis and release of DPP-4 are still incompletely understood. In adipose tissue, *Dpp4* expression is related to adipocyte size, and both insulin and the inflammatory cytokine TNF-α increase shedding of soluble DPP-4 from the membrane (79). Weight gain is also associated with greater *Dpp4* expression in the liver of mice prone to diet-induced obesity, independently of the degree of intra-hepatic fat accumulation (87). Furthermore, hepatocyte *Dpp4* expression is increased following exposure to high glucose (although not to insulin) (85), with weight gain further enhancing glucose-induced transcription of hepatic *Dpp4* (87). Accordingly, excess DPP-4, derived from adipocytes and/or hepatocytes, may act as a local mediator of inflammation and adipose/hepatic tissue insulin resistance, thereby forming one link between obesity and the pathogenesis of T2DM and metabolic disease (Figure 3).

## Pancreatic DPP-4

As well as influencing glucose homeostasis through its more widespread systemic effects, as discussed above, there is some evidence to suggest that DPP-4 may have local actions in the endocrine pancreas. Reports that DPP-4 may be associated with glucagon in the secretory granules of the pancreatic α-cell first emerged in 1993, when positive staining in pig islets was shown using immunohistochemical and enzyme histochemical techniques (96), but the pancreatic localization of DPP-4 has also been unequivocally demonstrated more recently using molecular biological techniques (97–99). It has also been suggested that DPP-4 might be secreted into the interstitial spaces within the islets (100) and, accordingly, DPP-4 activity can be measured in the media of human islet incubations (101), but whether this is simple shedding of membrane-associated DPP-4 or regulated secretion is not known. There appear to be species differences in pancreatic *Dpp4* expression; it is associated predominantly with the β-cell in rodents but with the α-cell in pig islets (102). The situation in human islets is less clear. The majority of studies have found DPP-4 to be associated primarily with the α-cell (97, 99, 102, 103), and to be present in only a subset of human β-cells (99), although one study reported that it was the β-cell which was the predominant site of expression (98). Moreover, its expression appears to be influenced by metabolic stress, with it being increased in islets from diet-induced obese mice compared to normal weight animals (104), but lower in human islets from diabetic compared to non-diabetic donors (98, 99, 104).

There is also some evidence that a local GLP-1 system may exist within the pancreas. Thus, several studies have demonstrated the presence of prohormone convertase 1/3 [PC1/3, the processing enzyme that cleaves proglucagon to generate GLP-1 (105)] and fully processed GLP-1 in rodent and human α-cells and islets in culture (106–110), and GLP-1 can be detected in the media, suggesting that it is secreted from the α-cells. Adult α-cells are not thought to produce much GLP-1, and a release of GLP-1 is barely detectable from the perfused pancreas of normal mice (111). Nevertheless, exposure to metabolic stress may be able to influence pancreatic GLP-1 expression. Thus, incubating human and rodent islets under high glucose conditions results in increased expression of PC1/3 and increased secretion of GLP-1 (101, 108–110), while exposure of α-cells to the proinflammatory cytokine, interleukin-6, also increased GLP-1 secretion (112). Moreover, the development of diabetes has been associated with greater pancreatic GLP-1 expression. Thus, the tissue content of GLP-1 is increased in islets from diabetic rodents (106, 110), and when cultured, islets obtained from human donors with T2DM or from diabetic rodents release more GLP-1 *ex vivo* than non-diabetic islets (98, 109, 113). It has, therefore, been suggested that metabolic stress may influence the pancreatic processing of proglucagon toward the production of GLP-1 as a compensatory response to help regulate glucose homeostasis and maintain β-cell function and survival (109, 113). In line with this, mice with an α-cell specific knock-down of PC1/3, resulting in significantly reduced islet GLP-1 content, were able to maintain normal glucose tolerance, suggesting that islet GLP-1 may not be essential and/or that it can be compensated for by intestinally derived GLP-1 under normal conditions. In contrast, when placed under metabolic stress with increased demand on the β-cell (induced by high fat feeding and a low dose of the β-cell toxin, streptozotocin), glucose tolerance did become impaired, supporting the view that paracrine GLP-1 may be required to maintain glucose homeostasis during adaptation to metabolic stress (114). On the other hand, it has also been suggested that pancreatic-derived GLP-1 may play a role even under normal circumstances. In proglucagon gene (*Gcg*) knockout mice, the GLP-1 receptor antagonist, exendin 9-39, led to impaired glucose tolerance when *Gcg* was reactivated in the pancreas, but not when intestinal *Gcg* was reactivated (115). This was taken to indicate that locally produced GLP-1 within the pancreas may be more important than its systemic effects for maintaining normal β-cell function. However, glucagon itself has recently been shown to be able to stimulate insulin secretion from the perfused mouse pancreas by a mechanism which appears to involve an interaction with GLP-1 receptors (since the effect was attenuated by exendin 9-39) (111), complicating assessment of the relative importance of any locally produced GLP-1.

Nevertheless, when taken together, these studies do raise the possibility that pancreatic DPP-4 may be able to influence islet function by modulating the activity of locally produced GLP-1. Accordingly, culturing human islets in the presence of a DPP-4 inhibitor resulted in increased levels of intact GLP-1 (98, 101, 104), increased insulin secretion (98, 99, 101, 104), and improved β-cell survival (99, 101). However, there is also a possibility that some of the effect may be independent of GLP-1, because DPP-4 inhibition still reduced β-cell apoptosis in human islets cultured in the presence of exendin 9-39 (99), suggesting that other mediators might be involved, but it was also suggested that an anti-inflammatory action could have played a role (99).

Collectively, the results discussed above could be interpreted to suggest that conditions likely to result in β-cell stress, such as obesity, hyperglycaemia, and inflammation, activate compensatory responses in an attempt to improve β-cell function and survival. In the α-cell, PC1/3 is up-regulated, resulting in increased processing of proglucagon to GLP-1 while DPP-4 is down-regulated in order to reduce degradation and enhance local intact GLP-1 concentrations. Whilst attractive as a theory, it should, nevertheless, be borne in mind that the majority of the studies cited above have been carried out in rodents or have examined isolated islets/α-cells under *in vitro* or *ex vivo* conditions, and whether a local pancreatic DPP-4/GLP-1 system plays an important role in normal human physiology or in the pharmacological effect of DPP-4 inhibitors is not clear.

## DPP-4 Inhibitors

As mentioned above, DPP-4 inhibitors are now an established and successful class of oral anti-hyperglycaemic agents, and are firmly embedded in guidelines for treating T2DM patients without atherosclerotic cardiovascular (CV) disease (116, 117). They are all small molecules which act as reversible competitive inhibitors of the catalytic activity of DPP-4 and, when used at the appropriate therapeutic dose, are associated with at least 70% inhibition of plasma DPP-4 activity (35) (although the actual extent of inhibition *in vivo* is likely to be higher, due to dilution of the inhibitor in the *ex vivo* assay). As discussed, the anti-hyperglycaemic effects of DPP-4 inhibition are mediated via enhancing levels of endogenous DPP-4 substrates, since the inhibitors themselves do not possess any inherent glucose-lowering activity. Accordingly, the degree of DPP-4 inhibition attained *in vivo* is sufficient to increase circulating levels of intact (active) incretins by 2- to 4-fold and result in improved glycaemic control (118). The risk of hypoglycaemia is not increased because the glucose-lowering effects of DPP-4 inhibition are self-limiting. Thus, the enzyme cannot be inhibited by more than 100% and the actions of the incretin hormones are glucose-dependent (16), meaning that insulin secretion is only stimulated when glucose levels rise above fasting levels.

Despite the theoretical potential for multiple endogenous DPP-4 substrates to be affected and for the DPP-4 protein to have diverse actions, as discussed in the sections above, pooled safety analyses (119–123), and large cardiovascular (CV) safety outcome trials (124–127) with the individual drugs, which between them have included many thousands of patients (Table 2), have uniformly shown that DPP-4 inhibitors are well-tolerated and not generally associated with adverse effects.

**Table 2:** Key published studies demonstrating the safety and tolerability of DPP-4 inhibitors.

**Inhibitor**	**Alogliptin**	**Linagliptin**	**Saxagliptin**	**Sitagliptin**	**Vildagliptin**
**POOLED SAFETY ANALYSES**					
References	Prately et al. (119)	Lehrke et al. (120)	Hirshberg et al. (121)	Engel et al. (122)	Schweizer et al. (123)
Study	6 phase 2 and 3 clinical trials	22 phase 1, 2 and 3 clinical trials	20 phase 2 and 3 clinical trials	25 phase 2 and 3 clinical trials	38 phase 2 and 3 clinical trials
Number	2,366	7,400	9,156	14,611	12,326
Comparator	Placebo	Placebo	Placebo or active comparator	Placebo or active comparator	Placebo or active comparator
Duration	12–26 weeks	< 2–104 weeks	4–206 weeks	12–104 weeks	12–104 weeks
**CARDIOVASCULAR SAFETY OUTCOME TRIALS**					
References	White et al. (124)	Rosenstock et al. (125)	Scirica et al. (127)	Green et al. (126)	–
Trial name	EXAMINE	CARMELINA	SAVOR-TIMI	TECOS	–
History of CV disease (%)	!00 (ACS)	57	78	100	–
Number	5,380	6,979	16,492	14,671	–
Comparator	Placebo	Placebo	Placebo	Placebo	–
Follow-up (y)	1.5	2.2	2.1	3.0	–
MACE HR (95% CI)	0.96 (upper, 1.16)	1.02 (0.89; 1.17)	1.00 (0.89; 1.12)	0.98 (0.88; 1.09)	–

*Pooled safety analyses examined patient-level safety data from phase 1–3 clinical trials in patients with T2DM. Cardiovascular safety outcome trials included patients with T2DM and either established CV disease or multiple CV risk factors (and albuminuria or impaired renal function in CARMELINA). The primary endpoint in the CV safety outcome trials was a composite of CV death, non-fatal myocardial infarct, and non-fatal stroke (and unstable angina requiring hospitalization in TECOS). Note: there is no equivalent large CV safety outcome trial with vildagliptin*.

*ACS, acute coronary syndrome; CI, confidence interval; CV, cardiovascular; HR, hazard ratio; MACE, major adverse cardiovascular events*.

There had been some initial questions over whether DPP-4 inhibition might compromise immune function, given the role of DPP-4/CD26 in the regulation of T-cell activity. However, neither the innate nor the adaptive immune response is affected by the DPP-4 inhibitors being used clinically (128), and reassuringly, there is no evidence for any increased risk of infections in the pooled safety analyses (119–123) or in the CV safety outcome trials (125–127), in line with the view that the catalytic activity of DPP-4 is not necessary for the co-stimulatory role of CD26 in T-cell activation. Similarly, these studies (Table 2) have not raised concern over increased cancer risk. However, in the CV safety outcome trials, a numerical imbalance in adjudicated cases of acute pancreatitis was noted (124–127, 129, 130), although the absolute risk was still very low. Thus, although the independent audit of all data carried out by regulatory authorities in the US and EU did not find evidence to support a causal relationship between incretin therapies and pancreatitis (131), it cannot be ruled out that a small increased risk of acute pancreatitis may exist in susceptible patients treated with DPP-4 inhibitors. The DPP-4 inhibitor CV safety outcome trials have unequivocally demonstrated that DPP-4 inhibition is not associated with any increased risk of major adverse CV events (MACE), with the four trials so far published (124–127) having hazard ratios (HR) for the primary outcome of between 0.96 and 1.02 (Table 2). Unexpectedly, hospitalization for heart failure was modestly increased in the SAVOR-TIMI trial with saxagliptin [3.5 vs. 2.8% for placebo, HR = 1.27 (127)]. Heart failure events were not increased in EXAMINE [alogliptin, HR = 1.07 (124)], TECOS [sitagliptin; HR = 1.00 (126)], or CARMELINA [linagliptin; HR = 0.90 (125)], even in those patients with previous heart failure (132–134), suggesting that the increased risk with saxagliptin is not mechanistically related to the inhibition of DPP-4 *per se*, but the cause is still unexplained. It has been suggested that it might simply be a chance finding or be related to the specific characteristics of the patients enrolled in the SAVOR-TIMI trial. However, it cannot be excluded that it could also be associated with some property of the saxagliptin molecule, unrelated to its action as a DPP-4 inhibitor. Accordingly, *in vitro* and *ex vivo* studies have shown that exposure to saxagliptin (135, 136) but not sitagliptin (136), results in altered cardiac electrophysiology and is associated with impaired cardiomyocyte contractility. The mechanism has been suggested to involve inhibition of cardiac intracellular signaling pathways (Ca^2+^/calmodulin-dependent protein kinase II and protein kinase C) due to saxagliptin-mediated inhibition DPP-9 (136). DPP-9 belongs to the same family as DPP-4, and it is known that saxagliptin is a less selective inhibitor, whereas sitagliptin is highly selective for DPP-4 (35) and does not alter DPP-9 activity (136). Nevertheless, although off-target inhibition of DPP-9 could be one potential explanation for the association between saxagliptin and heart failure in an experimental setting, it is uncertain whether mean exposure levels of saxagliptin reach a high enough level to affect DPP-9 activity when used at its therapeutic dose in humans.

Taken together, these pooled safety analyses (119–123) and CV outcome safety trials (124–127), together with review of published data from numerous clinical trials (39), provide reassuring evidence that the catalytic activity of the DPP-4 molecule can be modulated to enhance the beneficial metabolic effects of its substrates (such as the incretin hormones) without adversely affecting any of the protease-independent metabolic effects discussed above, or interfering with any of its other (non-metabolic) functions.

## Conclusions

Once known primarily as a marker of activated T-cells and associated with immune regulation, signal transduction, and apoptosis, it is now more generally recognized that the actions of DPP-4 may be more widespread, and that it has the potential, either directly or indirectly, to influence metabolic processes. The catalytic activity of the molecule appears to be involved in at least some of these actions, and several endogenous substrates have been identified, although a role for protease-independent functions related to metabolic homeostasis has also been suggested. However, while the relative importance of enzymatic vs. non-enzymatic actions and the overall significance of DPP-4 for normal physiological regulation in humans and for the pathogenesis of metabolic disease are still largely unknown, targeting the catalytic activity has been harnessed pharmacologically, and DPP-4 inhibitors now play an important part in the therapy of T2DM.

## Author Contributions

The author confirms being the sole contributor of this work and has approved it for publication.

### Conflict of Interest Statement

The author declares that the research was conducted in the absence of any commercial or financial relationships that could be construed as a potential conflict of interest.

## Abbreviations

BMI: body mass index
DPP: dipeptidyl peptidase
DPL: DPP4-like protein
FAP: fibroblast activation protein
GIP: glucose-dependent insulinotropic polypeptide
GLP-1: glucagon-like peptide-1
QPP: quiescent cell proline dipeptidase
SDF-1α: Stromal cell-derived factor-1α
T2DM: type 2 diabetes mellitus.
