# Corrigendum: Physiology and Pharmacology of DPP-4 in Glucose Homeostasis and the Treatment of Type 2 Diabetes

**DOI:** 10.3389/fendo.2019.00275

**Published:** 2019-05-03

**Authors:** Carolyn F. Deacon

**Affiliations:** Department of Biomedical Sciences, University of Copenhagen, Copenhagen, Denmark

**Keywords:** dipeptidyl peptidase-4, glucagon-like peptide-1, incretin, peptide degradation, therapy, type 2 diabetes

In the original article, there was a mistake in Table 2 “Key published studies demonstrating the safety and tolerability of DPP-4 inhibitors” as published. Unfortunately, two of the citations were transposed in the table. The correct citation for the SAVOR-TIMI trial is Scirica et al. (2013): Saxagliptin and cardiovascular outcomes in patients with type 2 diabetes mellitus. N Engl J Med 369: 1317–1326 (ref 127), while the correct citation for the TECOS trial is Green et al. (2015): Effect of sitagliptin on cardiovascular outcomes in type 2 diabetes. N Engl J Med 373: 232–242, 2015 (ref 126). The corrected Table 2 appears below. The authors apologize for this error and state that this does not change the scientific conclusions of the article in any way. The original article has been updated.

**Table 2:** Key published studies demonstrating the safety and tolerability of DPP-4 inhibitors.

**Inhibitor**	**Alogliptin**	**Linagliptin**	**Saxagliptin**	**Sitagliptin**	**Vildagliptin**
**POOLED SAFETY ANALYSES**					
References	Prately et al. (119)	Lehrke et al. (120)	Hirshberg et al. (121)	Engel et al. (122)	Schweizer et al. (123)
Study	6 phase 2 and 3 clinical trials	22 phase 1, 2 and 3 clinical trials	20 phase 2 and 3 clinical trials	25 phase 2 and 3 clinical trials	38 phase 2 and 3 clinical trials
Number	2,366	7,400	9,156	14,611	12,326
Comparator	Placebo	Placebo	Placebo or active comparator	Placebo or active comparator	Placebo or active comparator
Duration	12–26 weeks	< 2–104 weeks	4–206 weeks	12–104 weeks	12–104 weeks
**CARDIOVASCULAR SAFETY OUTCOME TRIALS**					
References	White et al. (124)	Rosenstock et al. (125)	Scirica et al. (127)	Green et al. (126)	–
Trial name	EXAMINE	CARMELINA	SAVOR-TIMI	TECOS	–
History of CV disease (%)	!00 (ACS)	57	78	100	–
Number	5,380	6,979	16,492	14,671	–
Comparator	Placebo	Placebo	Placebo	Placebo	–
Follow-up (y)	1.5	2.2	2.1	3.0	–
MACE HR (95% CI)	0.96 (upper, 1.16)	1.02 (0.89; 1.17)	1.00 (0.89; 1.12)	0.98 (0.88; 1.09)	–

*Pooled safety analyses examined patient-level safety data from phase 1–3 clinical trials in patients with T2DM. Cardiovascular safety outcome trials included patients with T2DM and either established CV disease or multiple CV risk factors (and albuminuria or impaired renal function in CARMELINA). The primary endpoint in the CV safety outcome trials was a composite of CV death, non-fatal myocardial infarct, and non-fatal stroke (and unstable angina requiring hospitalization in TECOS). Note: there is no equivalent large CV safety outcome trial with vildagliptin*.

*ACS, acute coronary syndrome; CI, confidence interval; CV, cardiovascular; HR, hazard ratio; MACE, major adverse cardiovascular events*.

